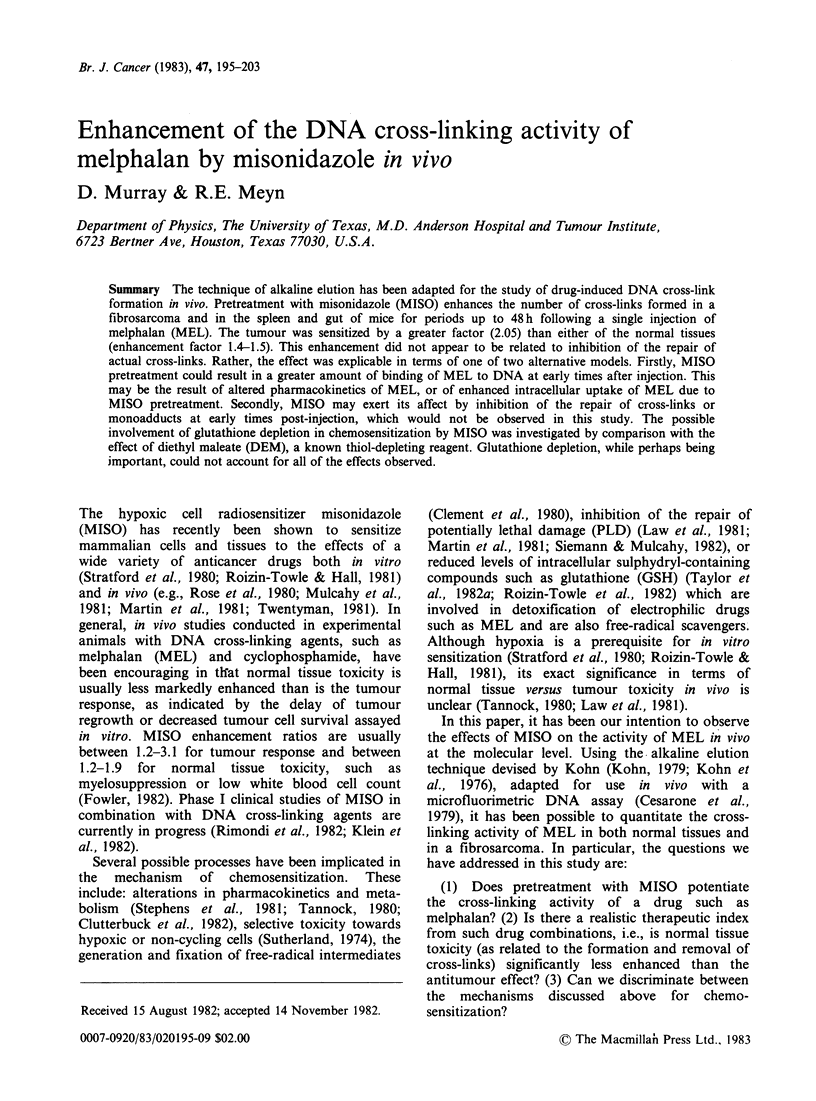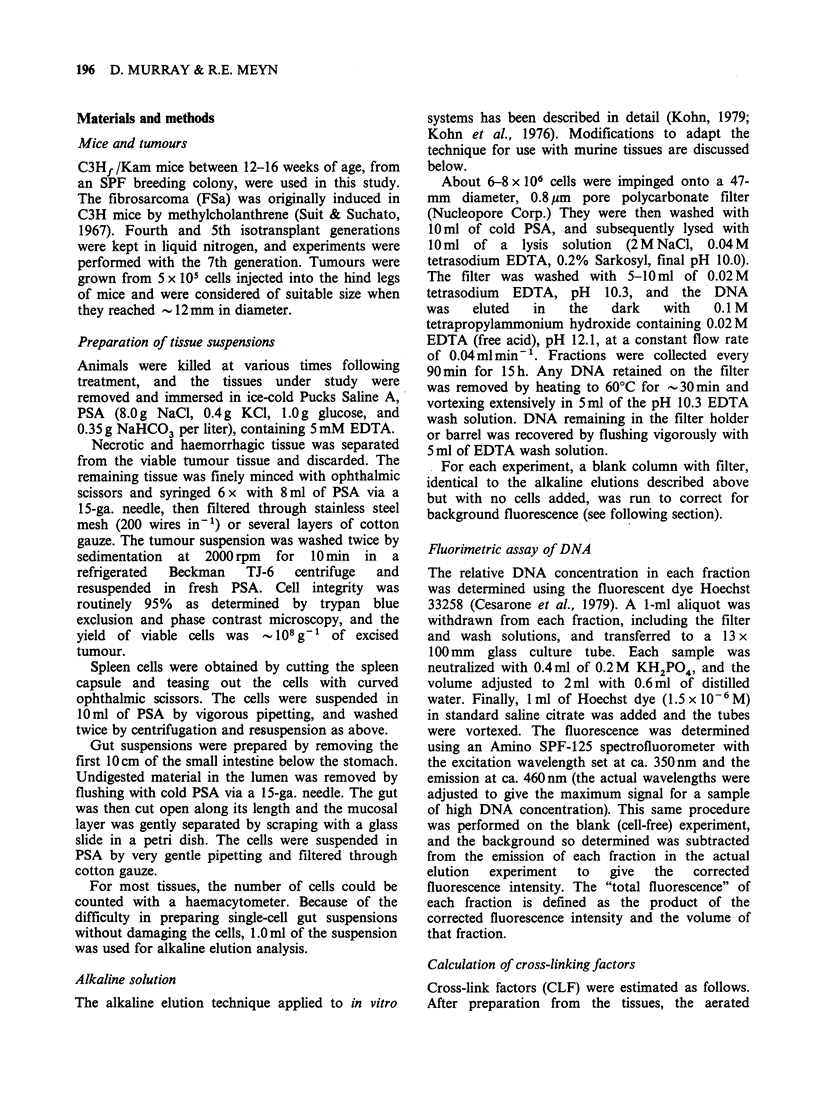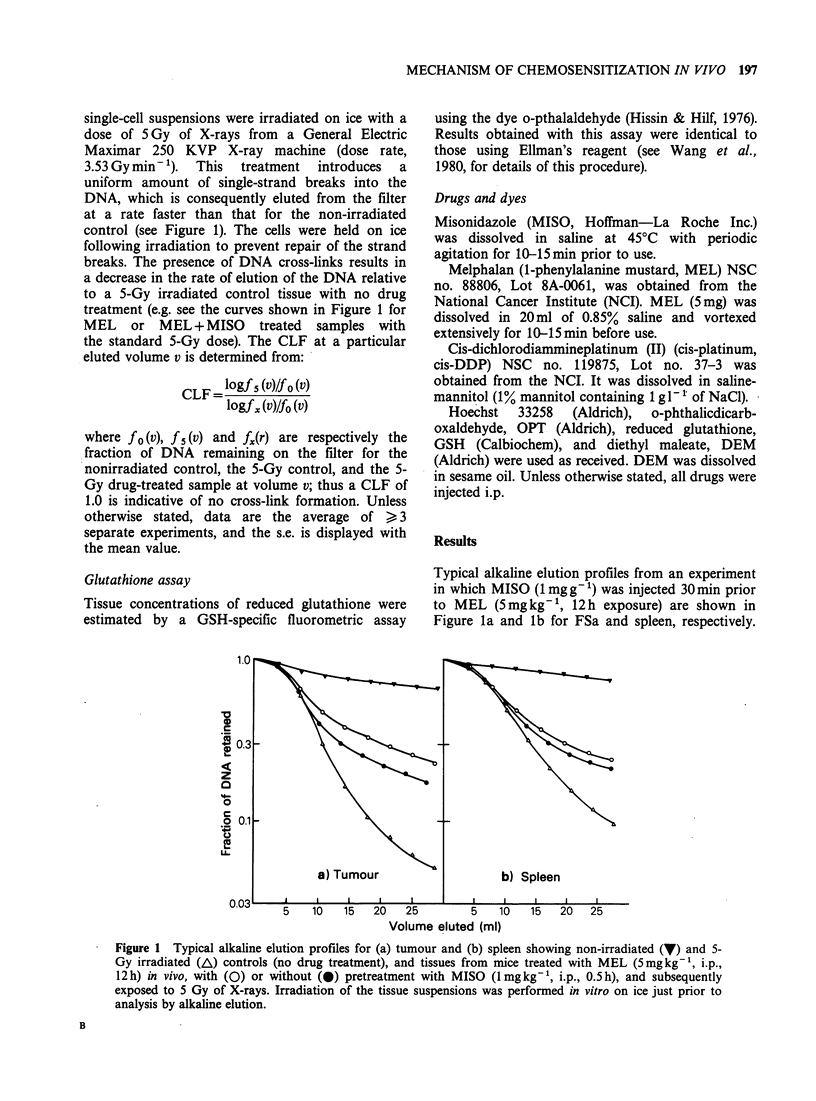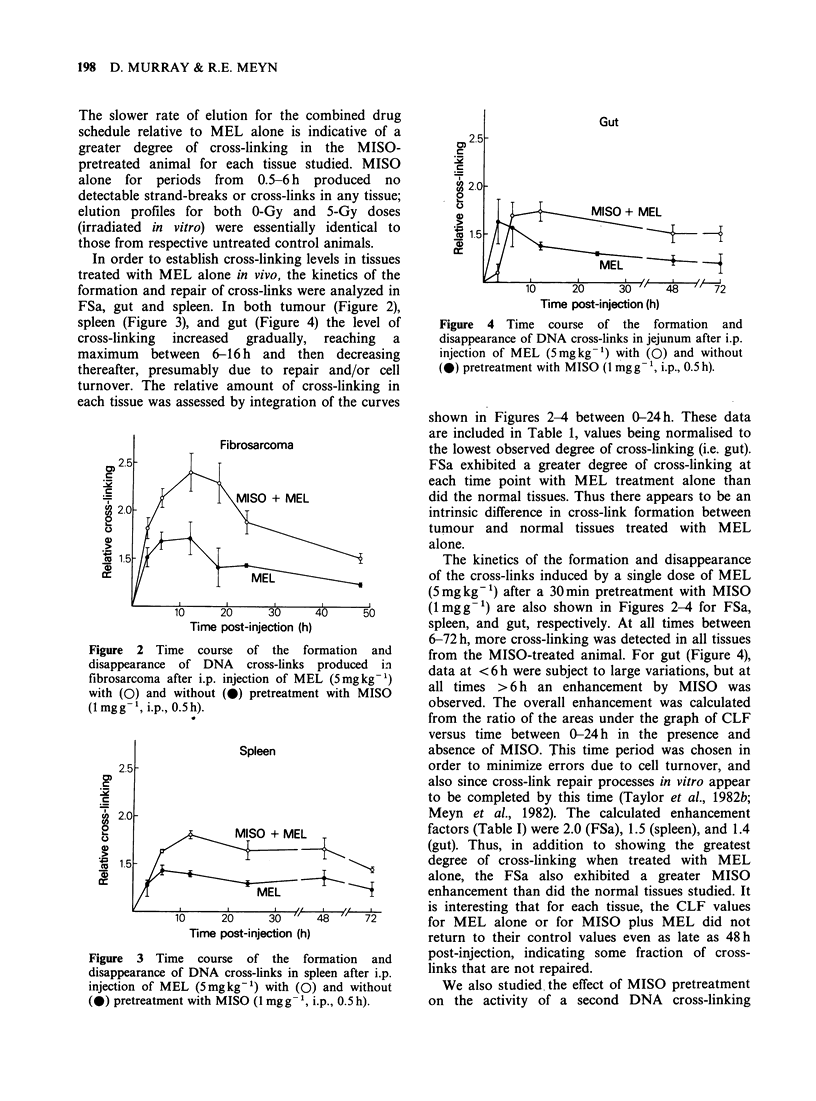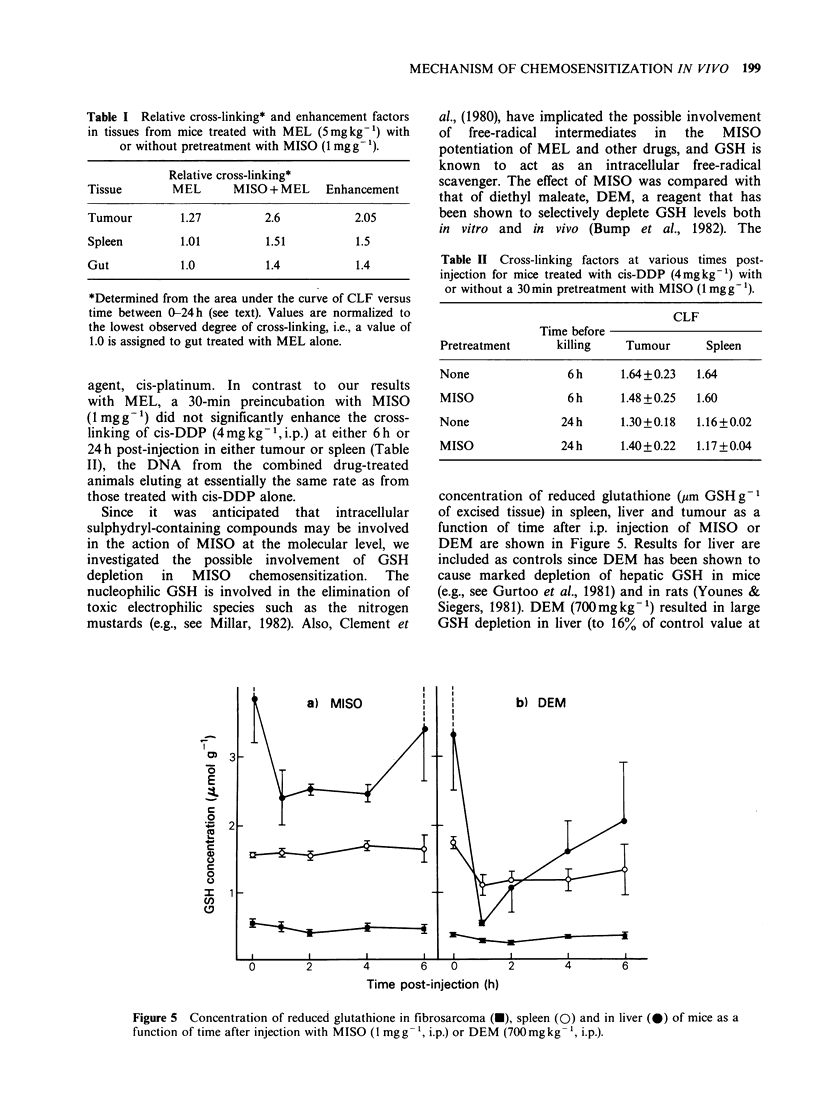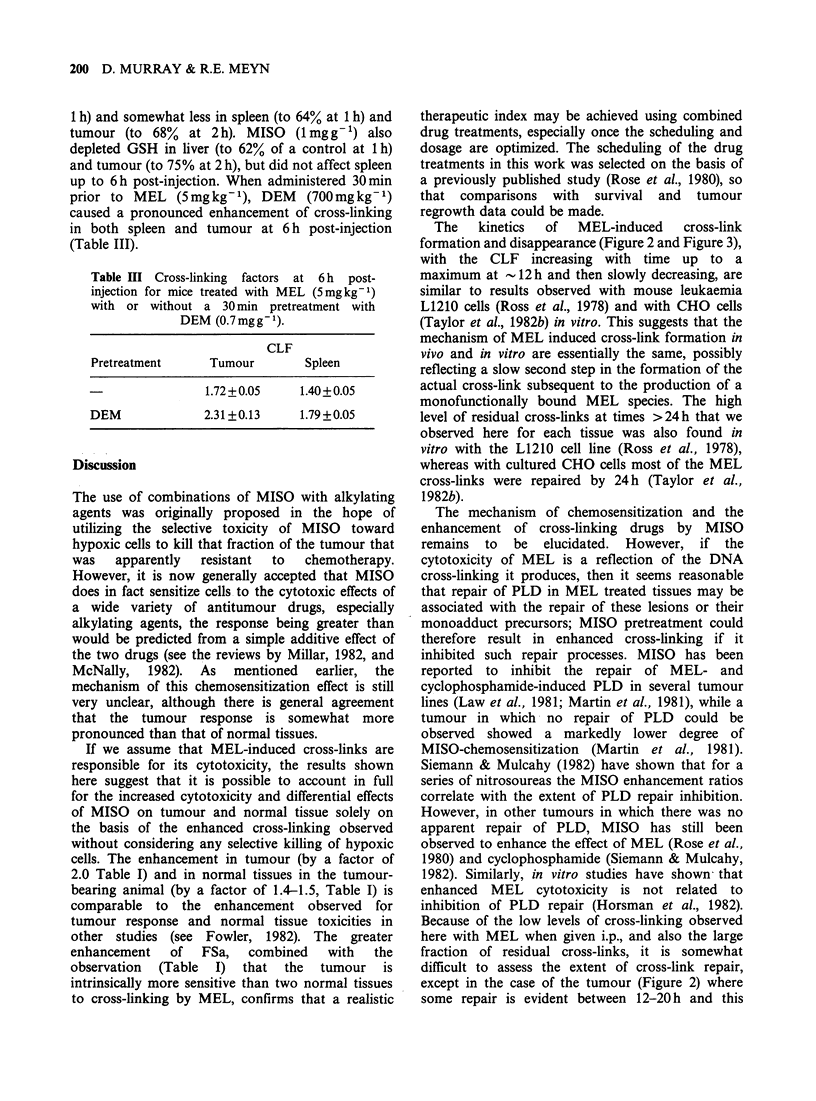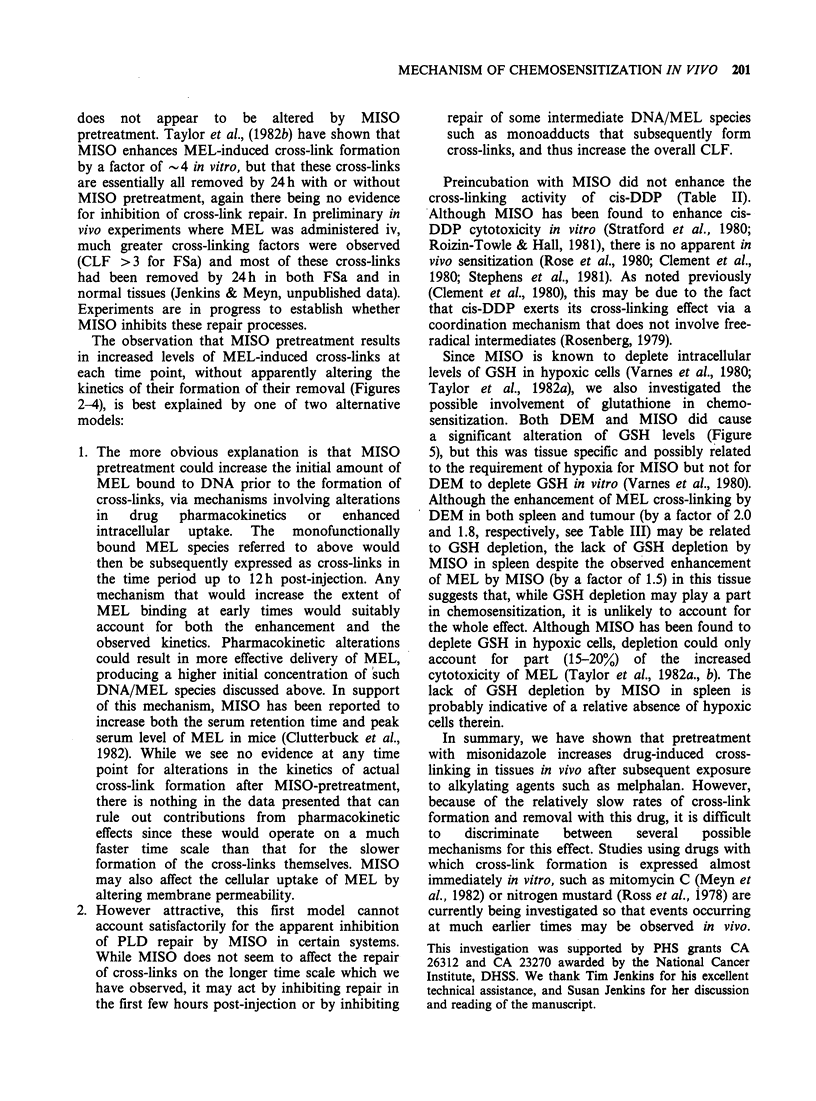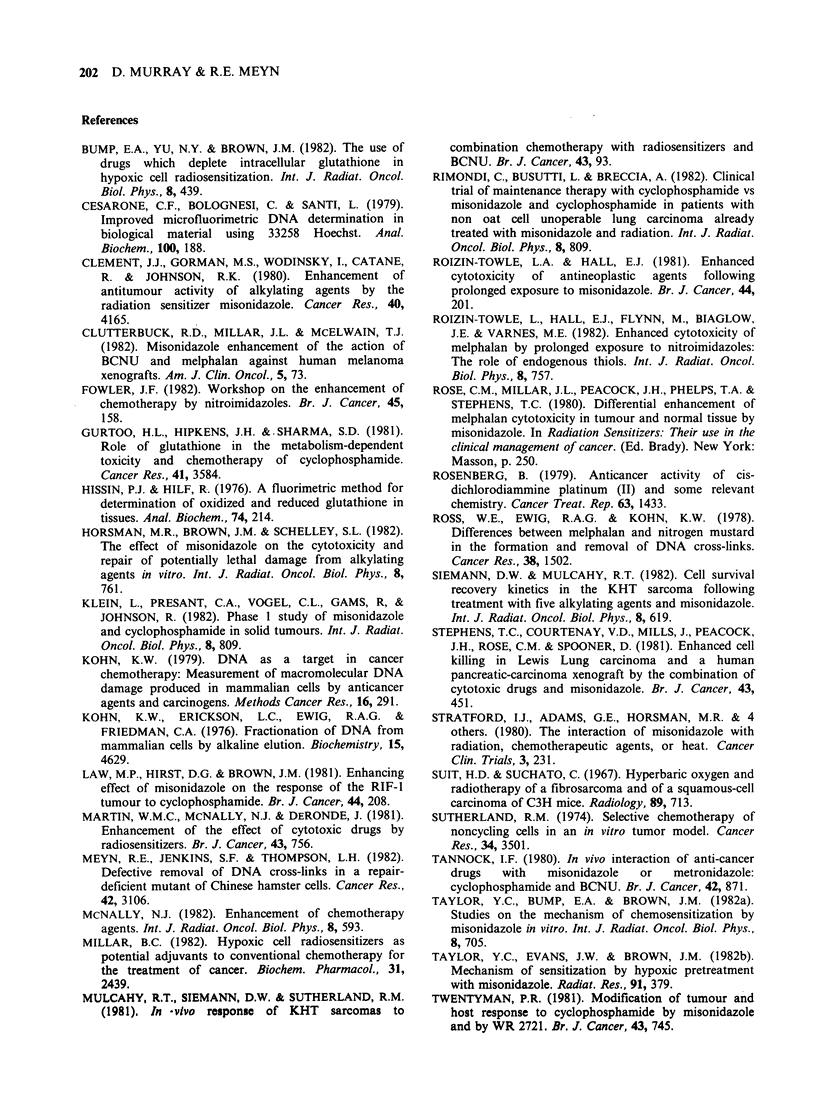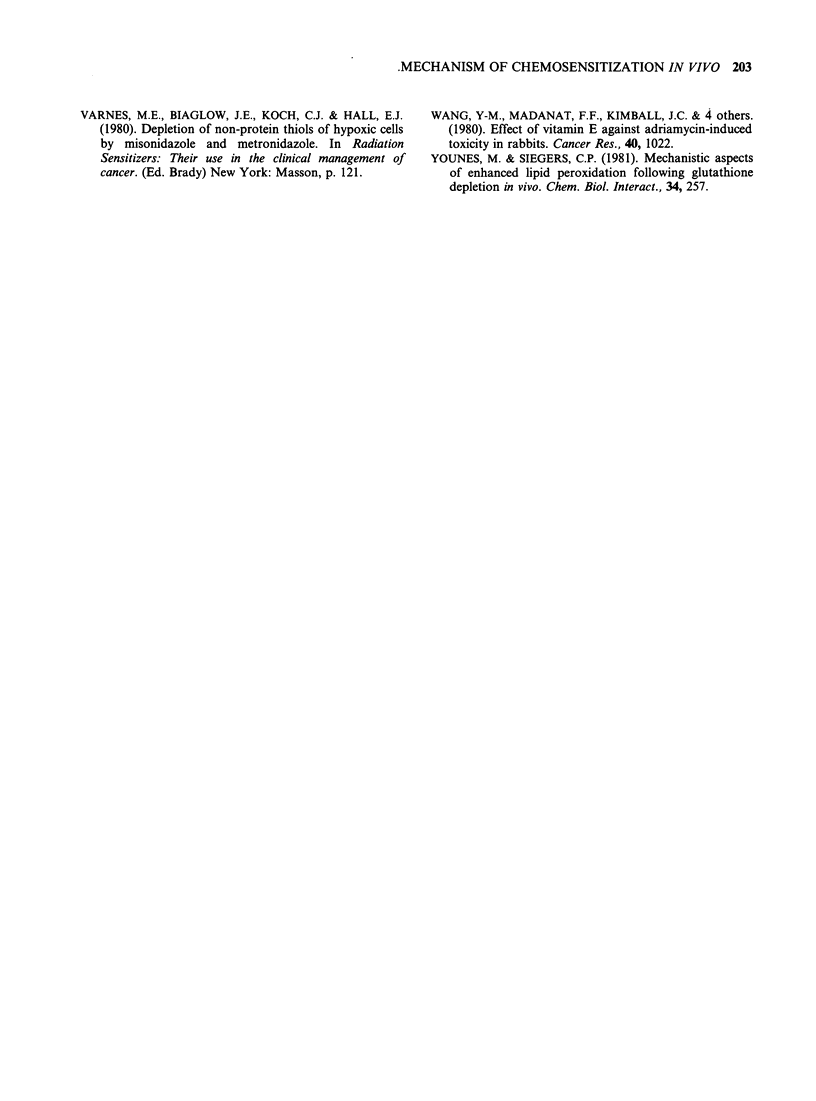# Enhancement of the DNA cross-linking activity of melphalan by misonidazole in vivo.

**DOI:** 10.1038/bjc.1983.27

**Published:** 1983-02

**Authors:** D. Murray, R. E. Meyn

## Abstract

The technique of alkaline elution has been adapted for the study of drug-induced DNA cross-link formation in vivo. Pretreatment with misonidazole (MISO) enhances the number of cross-links formed in a fibrosarcoma and in the spleen and gut of mice for periods up to 48 h following a single injection of melphalan (MEL). The tumour was sensitized by a greater factor (2.05) than either of the normal tissues (enhancement factor 1.4-1.5). This enhancement did not appear to be related to inhibition of the repair of actual cross-links. Rather, the effect was explicable in terms of one of two alternative models. Firstly, MISO pretreatment could result in a greater amount of binding of MEL to DNA at early times after injection. This may be the result of altered pharmacokinetics of MEL, or of enhanced intracellular uptake of MEL due to MISO pretreatment. Secondly, MISO may exert its affect by inhibition of the repair of cross-links or monoadducts at early times post-injection, which would not be observed in this study. The possible involvement of glutathione depletion in chemosensitization by MISO was investigated by comparison with the effect of diethyl maleate (DEM), a known thiol-depleting reagent. Glutathione depletion, while perhaps being important, could not account for all of the effects observed.


Br. J. Cancer (1983), **47**, 195–203

### Enhancement of the DNA cross-linking activity of melphalan by misonidazole *in vivo*

D. Murray & R.E. Meyn

*Department of Physics, The University of Texas, M.D. Anderson Hospital and Tumour Institute, 6723 Bertner Ave, Houston, Texas 77030, U.S.A.*

**Summary** The technique of alkaline elution has been adapted for the study of drug-induced DNA cross-link formation *in vivo*. Pretreatment with misonidazole (MISO) enhances the number of cross-links formed in a fibrosarcoma and in the spleen and gut of mice for periods up to 48 h following a single injection of melphalan (MEL). The tumour was sensitized by a greater factor (2.05) than either of the normal tissues (enhancement factor 1.4–1.5). This enhancement did not appear to be related to inhibition of the repair of actual cross-links. Rather, the effect was explicable in terms of one of two alternative models. Firstly, MISO pretreatment could result in a greater amount of binding of MEL to DNA at early times after injection. This may be the result of altered pharmacokinetics of MEL, or of enhanced intracellular uptake of MEL due to MISO pretreatment. Secondly, MISO may exert its affect by inhibition of the repair of cross-links or monoadducts at early times post-injection, which would not be observed in this study. The possible involvement of glutathione depletion in chemosensitization by MISO was investigated by comparison with the effect of diethyl maleate (DEM), a known thiol-depleting reagent. Glutathione depletion, while perhaps being important, could not account for all of the effects observed.

The hypoxic cell radiosensitizer misonidazole (MISO) has recently been shown to sensitize mammalian cells and tissues to the effects of a wide variety of anticancer drugs both *in vitro* (Stratford *et al.*, 1980; Roizin-Towle & Hall, 1981) and *in vivo* (e.g., Rose *et al.*, 1980; Mulcahy *et al.*, 1981; Martin *et al.*, 1981; Twentyman, 1981). In general, *in vivo* studies conducted in experimental animals with DNA cross-linking agents, such as melphalan (MEL) and cyclophosphamide, have been encouraging in that normal tissue toxicity is usually less markedly enhanced than is the tumour response, as indicated by the delay of tumour regrowth or decreased tumour cell survival assayed *in vitro*. MISO enhancement ratios are usually between 1.2–3.1 for tumour response and between 1.2–1.9 for normal tissue toxicity, such as myelosuppression or low white blood cell count (Fowler, 1982). Phase I clinical studies of MISO in combination with DNA cross-linking agents are currently in progress (Rimondi *et al.*, 1982; Klein *et al.*, 1982).

Several possible processes have been implicated in the mechanism of chemosensitization. These include: alterations in pharmacokinetics and metabolism (Stephens *et al.*, 1981; Tannock, 1980; Clutterbuck *et al.*, 1982), selective toxicity towards hypoxic or non-cycling cells (Sutherland, 1974), the generation and fixation of free-radical intermediates (Clement *et al.*, 1980), inhibition of the repair of potentially lethal damage (PLD) (Law *et al.*, 1981; Martin *et al.*, 1981; Siemann & Mulcahy, 1982), or reduced levels of intracellular sulphydryl-containing compounds such as glutathione (GSH) (Taylor *et al.*, 1982*a*; Roizin-Towle *et al.*, 1982) which are involved in detoxification of electrophilic drugs such as MEL and are also free-radical scavengers. Although hypoxia is a prerequisite for *in vitro* sensitization (Stratford *et al.*, 1980; Roizin-Towle & Hall, 1981), its exact significance in terms of normal tissue *versus* tumour toxicity *in vivo* is unclear (Tannock, 1980; Law *et al.*, 1981).

In this paper, it has been our intention to observe the effects of MISO on the activity of MEL *in vivo* at the molecular level. Using the alkaline elution technique devised by Kohn (Kohn, 1979; Kohn *et al.*, 1976), adapted for use *in vivo* with a microfluorimetric DNA assay (Cesarone *et al.*, 1979), it has been possible to quantitate the cross-linking activity of MEL in both normal tissues and in a fibrosarcoma. In particular, the questions we have addressed in this study are:

(1) Does pretreatment with MISO potentiate the cross-linking activity of a drug such as melphalan? (2) Is there a realistic therapeutic index from such drug combinations, i.e., is normal tissue toxicity (as related to the formation and removal of cross-links) significantly less enhanced than the antitumour effect? (3) Can we discriminate between the mechanisms discussed above for chemosensitization?

Received 15 August 1982; accepted 14 November 1982.

0007-0920/83/020195-09 $02.00

© The Macmillan Press Ltd., 1983

### Materials and methods

### *Mice and tumours*

$C3H_f$/Kam mice between 12–16 weeks of age, from an SPF breeding colony, were used in this study. The fibrosarcoma (FSa) was originally induced in C3H mice by methylcholanthrene (Suit & Suchato, 1967). Fourth and 5th isotransplant generations were kept in liquid nitrogen, and experiments were performed with the 7th generation. Tumours were grown from $5 \times 10^5$ cells injected into the hind legs of mice and were considered of suitable size when they reached ~12 mm in diameter.

### *Preparation of tissue suspensions*

Animals were killed at various times following treatment, and the tissues under study were removed and immersed in ice-cold Pucks Saline A, PSA (8.0 g NaCl, 0.4 g KCl, 1.0 g glucose, and 0.35 g $NaHCO_3$ per liter), containing 5 mM EDTA.

Necrotic and haemorrhagic tissue was separated from the viable tumour tissue and discarded. The remaining tissue was finely minced with ophthalmic scissors and syringed 6 × with 8 ml of PSA via a 15-ga. needle, then filtered through stainless steel mesh (200 wires $in^{-1}$) or several layers of cotton gauze. The tumour suspension was washed twice by sedimentation at 2000 rpm for 10 min in a refrigerated Beckman TJ-6 centrifuge and resuspended in fresh PSA. Cell integrity was routinely 95% as determined by trypan blue exclusion and phase contrast microscopy, and the yield of viable cells was $\sim 10^8 g^{-1}$ of excised tumour.

Spleen cells were obtained by cutting the spleen capsule and teasing out the cells with curved ophthalmic scissors. The cells were suspended in 10 ml of PSA by vigorous pipetting, and washed twice by centrifugation and resuspension as above.

Gut suspensions were prepared by removing the first 10 cm of the small intestine below the stomach. Undigested material in the lumen was removed by flushing with cold PSA via a 15-ga. needle. The gut was then cut open along its length and the mucosal layer was gently separated by scraping with a glass slide in a petri dish. The cells were suspended in PSA by very gentle pipetting and filtered through cotton gauze.

For most tissues, the number of cells could be counted with a haemacytometer. Because of the difficulty in preparing single-cell gut suspensions without damaging the cells, 1.0 ml of the suspension was used for alkaline elution analysis.

### *Alkaline solution*

The alkaline elution technique applied to *in vitro* systems has been described in detail (Kohn, 1979; Kohn *et al.*, 1976). Modifications to adapt the technique for use with murine tissues are discussed below.

About $6–8 \times 10^6$ cells were impinged onto a 47-mm diameter, 0.8 μm pore polycarbonate filter (Nucleopore Corp.) They were then washed with 10 ml of cold PSA, and subsequently lysed with 10 ml of a lysis solution (2 M NaCl, 0.04 M tetrasodium EDTA, 0.2% Sarkosyl, final pH 10.0). The filter was washed with 5–10 ml of 0.02 M tetrasodium EDTA, pH 10.3, and the DNA was eluted in the dark with 0.1 M tetrapropylammonium hydroxide containing 0.02 M EDTA (free acid), pH 12.1, at a constant flow rate of 0.04 ml $min^{-1}$. Fractions were collected every 90 min for 15 h. Any DNA retained on the filter was removed by heating to 60°C for ~30 min and vortexing extensively in 5 ml of the pH 10.3 EDTA wash solution. DNA remaining in the filter holder or barrel was recovered by flushing vigorously with 5 ml of EDTA wash solution.

For each experiment, a blank column with filter, identical to the alkaline elutions described above but with no cells added, was run to correct for background fluorescence (see following section).

### *Fluorimetric assay of DNA*

The relative DNA concentration in each fraction was determined using the fluorescent dye Hoechst 33258 (Cesarone *et al.*, 1979). A 1-ml aliquot was withdrawn from each fraction, including the filter and wash solutions, and transferred to a 13 × 100 mm glass culture tube. Each sample was neutralized with 0.4 ml of 0.2 M $KH_2PO_4$, and the volume adjusted to 2 ml with 0.6 ml of distilled water. Finally, 1 ml of Hoechst dye ($1.5 \times 10^{-6}$ M) in standard saline citrate was added and the tubes were vortexed. The fluorescence was determined using an Amino SPF-125 spectrofluorometer with the excitation wavelength set at ca. 350 nm and the emission at ca. 460 nm (the actual wavelengths were adjusted to give the maximum signal for a sample of high DNA concentration). This same procedure was performed on the blank (cell-free) experiment, and the background so determined was subtracted from the emission of each fraction in the actual elution experiment to give the corrected fluorescence intensity. The "total fluorescence" of each fraction is defined as the product of the corrected fluorescence intensity and the volume of that fraction.

### *Calculation of cross-linking factors*

Cross-link factors (CLF) were estimated as follows. After preparation from the tissues, the aerated single-cell suspensions were irradiated on ice with a dose of 5 Gy of X-rays from a General Electric Maximar 250 KVP X-ray machine (dose rate, 3.53 Gy min$^{-1}$). This treatment introduces a uniform amount of single-strand breaks into the DNA, which is consequently eluted from the filter at a rate faster than that for the non-irradiated control (see Figure 1). The cells were held on ice following irradiation to prevent repair of the strand breaks. The presence of DNA cross-links results in a decrease in the rate of elution of the DNA relative to a 5-Gy irradiated control tissue with no drug treatment (e.g. see the curves shown in Figure 1 for MEL or MEL + MISO treated samples with the standard 5-Gy dose). The CLF at a particular eluted volume $v$ is determined from:

$$\mathrm{CLF} = \frac{\log f_5(v)/f_0(v)}{\log f_x(v)/f_0(v)}$$

where $f_0(v)$, $f_5(v)$ and $f_x(r)$ are respectively the fraction of DNA remaining on the filter for the nonirradiated control, the 5-Gy control, and the 5-Gy drug-treated sample at volume $v$; thus a CLF of 1.0 is indicative of no cross-link formation. Unless otherwise stated, data are the average of $\geqslant 3$ separate experiments, and the s.e. is displayed with the mean value.

*Glutathione assay*

Tissue concentrations of reduced glutathione were estimated by a GSH-specific fluorometric assay using the dye o-pthalaldehyde (Hissin & Hilf, 1976). Results obtained with this assay were identical to those using Ellman's reagent (see Wang *et al.*, 1980, for details of this procedure).

*Drugs and dyes*

Misonidazole (MISO, Hoffman—La Roche Inc.) was dissolved in saline at 45°C with periodic agitation for 10–15 min prior to use.

Melphalan (1-phenylalanine mustard, MEL) NSC no. 88806, Lot 8A-0061, was obtained from the National Cancer Institute (NCI). MEL (5 mg) was dissolved in 20 ml of 0.85% saline and vortexed extensively for 10–15 min before use.

Cis-dichlorodiammineplatinum (II) (cis-platinum, cis-DDP) NSC no. 119875, Lot no. 37–3 was obtained from the NCI. It was dissolved in saline-mannitol (1% mannitol containing 1 g l$^{-1}$ of NaCl).

Hoechst 33258 (Aldrich), o-phthalicdicarboxaldehyde, OPT (Aldrich), reduced glutathione, GSH (Calbiochem), and diethyl maleate, DEM (Aldrich) were used as received. DEM was dissolved in sesame oil. Unless otherwise stated, all drugs were injected i.p.

### Results

Typical alkaline elution profiles from an experiment in which MISO (1 mg g$^{-1}$) was injected 30 min prior to MEL (5 mg kg$^{-1}$, 12 h exposure) are shown in Figure 1a and 1b for FSa and spleen, respectively.

**Figure 1** Typical alkaline elution profiles for (a) tumour and (b) spleen showing non-irradiated (▼) and 5-Gy irradiated (△) controls (no drug treatment), and tissues from mice treated with MEL (5 mg kg$^{-1}$, i.p., 12 h) *in vivo*, with (○) or without (●) pretreatment with MISO (1 mg kg$^{-1}$, i.p., 0.5 h), and subsequently exposed to 5 Gy of X-rays. Irradiation of the tissue suspensions was performed *in vitro* on ice just prior to analysis by alkaline elution.

The slower rate of elution for the combined drug schedule relative to MEL alone is indicative of a greater degree of cross-linking in the MISO-pretreated animal for each tissue studied. MISO alone for periods from 0.5–6 h produced no detectable strand-breaks or cross-links in any tissue; elution profiles for both 0-Gy and 5-Gy doses (irradiated *in vitro*) were essentially identical to those from respective untreated control animals.

In order to establish cross-linking levels in tissues treated with MEL alone *in vivo*, the kinetics of the formation and repair of cross-links were analyzed in FSa, gut and spleen. In both tumour (Figure 2), spleen (Figure 3), and gut (Figure 4) the level of cross-linking increased gradually, reaching a maximum between 6–16 h and then decreasing thereafter, presumably due to repair and/or cell turnover. The relative amount of cross-linking in each tissue was assessed by integration of the curves

**Figure 2** Time course of the formation and disappearance of DNA cross-links produced in fibrosarcoma after i.p. injection of MEL (5 mg kg$^{-1}$) with (○) and without (●) pretreatment with MISO (1 mg g$^{-1}$, i.p., 0.5 h).

**Figure 3** Time course of the formation and disappearance of DNA cross-links in spleen after i.p. injection of MEL (5 mg kg$^{-1}$) with (○) and without (●) pretreatment with MISO (1 mg g$^{-1}$, i.p., 0.5 h).

**Figure 4** Time course of the formation and disappearance of DNA cross-links in jejunum after i.p. injection of MEL (5 mg kg$^{-1}$) with (○) and without (●) pretreatment with MISO (1 mg g$^{-1}$, i.p., 0.5 h).

shown in Figures 2–4 between 0–24 h. These data are included in Table 1, values being normalised to the lowest observed degree of cross-linking (i.e. gut). FSa exhibited a greater degree of cross-linking at each time point with MEL treatment alone than did the normal tissues. Thus there appears to be an intrinsic difference in cross-link formation between tumour and normal tissues treated with MEL alone.

The kinetics of the formation and disappearance of the cross-links induced by a single dose of MEL (5 mg kg$^{-1}$) after a 30 min pretreatment with MISO (1 mg g$^{-1}$) are also shown in Figures 2–4 for FSa, spleen, and gut, respectively. At all times between 6–72 h, more cross-linking was detected in all tissues from the MISO-treated animal. For gut (Figure 4), data at $<6$ h were subject to large variations, but at all times $>6$ h an enhancement by MISO was observed. The overall enhancement was calculated from the ratio of the areas under the graph of CLF versus time between 0–24 h in the presence and absence of MISO. This time period was chosen in order to minimize errors due to cell turnover, and also since cross-link repair processes *in vitro* appear to be completed by this time (Taylor *et al.*, 1982*b*; Meyn *et al.*, 1982). The calculated enhancement factors (Table I) were 2.0 (FSa), 1.5 (spleen), and 1.4 (gut). Thus, in addition to showing the greatest degree of cross-linking when treated with MEL alone, the FSa also exhibited a greater MISO enhancement than did the normal tissues studied. It is interesting that for each tissue, the CLF values for MEL alone or for MISO plus MEL did not return to their control values even as late as 48 h post-injection, indicating some fraction of cross-links that are not repaired.

We also studied the effect of MISO pretreatment on the activity of a second DNA cross-linking

**Table I** Relative cross-linking* and enhancement factors in tissues from mice treated with MEL (5 mg kg$^{-1}$) with or without pretreatment with MISO (1 mg g$^{-1}$).

| Tissue | Relative cross-linking* MEL | MISO+MEL | Enhancement |
|---|---|---|---|
| Tumour | 1.27 | 2.6 | 2.05 |
| Spleen | 1.01 | 1.51 | 1.5 |
| Gut | 1.0 | 1.4 | 1.4 |

*Determined from the area under the curve of CLF versus time between 0–24 h (see text). Values are normalized to the lowest observed degree of cross-linking, i.e., a value of 1.0 is assigned to gut treated with MEL alone.

agent, cis-platinum. In contrast to our results with MEL, a 30-min preincubation with MISO (1 mg g$^{-1}$) did not significantly enhance the cross-linking of cis-DDP (4 mg kg$^{-1}$, i.p.) at either 6 h or 24 h post-injection in either tumour or spleen (Table II), the DNA from the combined drug-treated animals eluting at essentially the same rate as from those treated with cis-DDP alone.

Since it was anticipated that intracellular sulphydryl-containing compounds may be involved in the action of MISO at the molecular level, we investigated the possible involvement of GSH depletion in MISO chemosensitization. The nucleophilic GSH is involved in the elimination of toxic electrophilic species such as the nitrogen mustards (e.g., see Millar, 1982). Also, Clement *et al.*, (1980), have implicated the possible involvement of free-radical intermediates in the MISO potentiation of MEL and other drugs, and GSH is known to act as an intracellular free-radical scavenger. The effect of MISO was compared with that of diethyl maleate, DEM, a reagent that has been shown to selectively deplete GSH levels both *in vitro* and *in vivo* (Bump *et al.*, 1982). The

**Table II** Cross-linking factors at various times post-injection for mice treated with cis-DDP (4 mg kg$^{-1}$) with or without a 30 min pretreatment with MISO (1 mg g$^{-1}$).

| Pretreatment | Time before killing | CLF Tumour | CLF Spleen |
|---|---|---|---|
| None | 6 h | 1.64 ± 0.23 | 1.64 |
| MISO | 6 h | 1.48 ± 0.25 | 1.60 |
| None | 24 h | 1.30 ± 0.18 | 1.16 ± 0.02 |
| MISO | 24 h | 1.40 ± 0.22 | 1.17 ± 0.04 |

concentration of reduced glutathione ($\mu$m GSH g$^{-1}$ of excised tissue) in spleen, liver and tumour as a function of time after i.p. injection of MISO or DEM are shown in Figure 5. Results for liver are included as controls since DEM has been shown to cause marked depletion of hepatic GSH in mice (e.g., see Gurtoo *et al.*, 1981) and in rats (Younes & Siegers, 1981). DEM (700 mg kg$^{-1}$) resulted in large GSH depletion in liver (to 16% of control value at

**Figure 5** Concentration of reduced glutathione in fibrosarcoma (■), spleen (○) and in liver (●) of mice as a function of time after injection with MISO (1 mg g$^{-1}$, i.p.) or DEM (700 mg kg$^{-1}$, i.p.).

1 h) and somewhat less in spleen (to 64% at 1 h) and tumour (to 68% at 2 h). MISO ($1 mg g^{-1}$) also depleted GSH in liver (to 62% of a control at 1 h) and tumour (to 75% at 2 h), but did not affect spleen up to 6 h post-injection. When administered 30 min prior to MEL ($5 mg kg^{-1}$), DEM ($700 mg kg^{-1}$) caused a pronounced enhancement of cross-linking in both spleen and tumour at 6 h post-injection (Table III).

**Table III** Cross-linking factors at 6 h post-injection for mice treated with MEL ($5 mg kg^{-1}$) with or without a 30 min pretreatment with DEM ($0.7 mg g^{-1}$).

| Pretreatment | CLF Tumour | CLF Spleen |
|---|---|---|
| — | 1.72 ± 0.05 | 1.40 ± 0.05 |
| DEM | 2.31 ± 0.13 | 1.79 ± 0.05 |

### Discussion

The use of combinations of MISO with alkylating agents was originally proposed in the hope of utilizing the selective toxicity of MISO toward hypoxic cells to kill that fraction of the tumour that was apparently resistant to chemotherapy. However, it is now generally accepted that MISO does in fact sensitize cells to the cytotoxic effects of a wide variety of antitumour drugs, especially alkylating agents, the response being greater than would be predicted from a simple additive effect of the two drugs (see the reviews by Millar, 1982, and McNally, 1982). As mentioned earlier, the mechanism of this chemosensitization effect is still very unclear, although there is general agreement that the tumour response is somewhat more pronounced than that of normal tissues.

If we assume that MEL-induced cross-links are responsible for its cytotoxicity, the results shown here suggest that it is possible to account in full for the increased cytotoxicity and differential effects of MISO on tumour and normal tissue solely on the basis of the enhanced cross-linking observed without considering any selective killing of hypoxic cells. The enhancement in tumour (by a factor of 2.0 Table I) and in normal tissues in the tumour-bearing animal (by a factor of 1.4–1.5, Table I) is comparable to the enhancement observed for tumour response and normal tissue toxicities in other studies (see Fowler, 1982). The greater enhancement of FSa, combined with the observation (Table I) that the tumour is intrinsically more sensitive than two normal tissues to cross-linking by MEL, confirms that a realistic therapeutic index may be achieved using combined drug treatments, especially once the scheduling and dosage are optimized. The scheduling of the drug treatments in this work was selected on the basis of a previously published study (Rose *et al.*, 1980), so that comparisons with survival and tumour regrowth data could be made.

The kinetics of MEL-induced cross-link formation and disappearance (Figure 2 and Figure 3), with the CLF increasing with time up to a maximum at ~12 h and then slowly decreasing, are similar to results observed with mouse leukaemia L1210 cells (Ross *et al.*, 1978) and with CHO cells (Taylor *et al.*, 1982*b*) *in vitro*. This suggests that the mechanism of MEL induced cross-link formation *in vivo* and *in vitro* are essentially the same, possibly reflecting a slow second step in the formation of the actual cross-link subsequent to the production of a monofunctionally bound MEL species. The high level of residual cross-links at times >24 h that we observed here for each tissue was also found *in vitro* with the L1210 cell line (Ross *et al.*, 1978), whereas with cultured CHO cells most of the MEL cross-links were repaired by 24 h (Taylor *et al.*, 1982*b*).

The mechanism of chemosensitization and the enhancement of cross-linking drugs by MISO remains to be elucidated. However, if the cytotoxicity of MEL is a reflection of the DNA cross-linking it produces, then it seems reasonable that repair of PLD in MEL treated tissues may be associated with the repair of these lesions or their monoadduct precursors; MISO pretreatment could therefore result in enhanced cross-linking if it inhibited such repair processes. MISO has been reported to inhibit the repair of MEL- and cyclophosphamide-induced PLD in several tumour lines (Law *et al.*, 1981; Martin *et al.*, 1981), while a tumour in which no repair of PLD could be observed showed a markedly lower degree of MISO-chemosensitization (Martin *et al.*, 1981). Siemann & Mulcahy (1982) have shown that for a series of nitrosoureas the MISO enhancement ratios correlate with the extent of PLD repair inhibition. However, in other tumours in which there was no apparent repair of PLD, MISO has still been observed to enhance the effect of MEL (Rose *et al.*, 1980) and cyclophosphamide (Siemann & Mulcahy, 1982). Similarly, *in vitro* studies have shown that enhanced MEL cytotoxicity is not related to inhibition of PLD repair (Horsman *et al.*, 1982). Because of the low levels of cross-linking observed here with MEL when given i.p., and also the large fraction of residual cross-links, it is somewhat difficult to assess the extent of cross-link repair, except in the case of the tumour (Figure 2) where some repair is evident between 12–20 h and this does not appear to be altered by MISO pretreatment. Taylor *et al.*, (1982*b*) have shown that MISO enhances MEL-induced cross-link formation by a factor of ~4 *in vitro,* but that these cross-links are essentially all removed by 24 h with or without MISO pretreatment, again there being no evidence for inhibition of cross-link repair. In preliminary *in vivo* experiments where MEL was administered iv, much greater cross-linking factors were observed (CLF >3 for FSa) and most of these cross-links had been removed by 24 h in both FSa and in normal tissues (Jenkins & Meyn, unpublished data). Experiments are in progress to establish whether MISO inhibits these repair processes.

The observation that MISO pretreatment results in increased levels of MEL-induced cross-links at each time point, without apparently altering the kinetics of their formation of their removal (Figures 2–4), is best explained by one of two alternative models:

1. The more obvious explanation is that MISO pretreatment could increase the initial amount of MEL bound to DNA prior to the formation of cross-links, via mechanisms involving alterations in drug pharmacokinetics or enhanced intracellular uptake. The monofunctionally bound MEL species referred to above would then be subsequently expressed as cross-links in the time period up to 12 h post-injection. Any mechanism that would increase the extent of MEL binding at early times would suitably account for both the enhancement and the observed kinetics. Pharmacokinetic alterations could result in more effective delivery of MEL, producing a higher initial concentration of such DNA/MEL species discussed above. In support of this mechanism, MISO has been reported to increase both the serum retention time and peak serum level of MEL in mice (Clutterbuck *et al.*, 1982). While we see no evidence at any time point for alterations in the kinetics of actual cross-link formation after MISO-pretreatment, there is nothing in the data presented that can rule out contributions from pharmacokinetic effects since these would operate on a much faster time scale than that for the slower formation of the cross-links themselves. MISO may also affect the cellular uptake of MEL by altering membrane permeability.
2. However attractive, this first model cannot account satisfactorily for the apparent inhibition of PLD repair by MISO in certain systems. While MISO does not seem to affect the repair of cross-links on the longer time scale which we have observed, it may act by inhibiting repair in the first few hours post-injection or by inhibiting repair of some intermediate DNA/MEL species such as monoadducts that subsequently form cross-links, and thus increase the overall CLF.

Preincubation with MISO did not enhance the cross-linking activity of cis-DDP (Table II). Although MISO has been found to enhance cis-DDP cytotoxicity *in vitro* (Stratford *et al.*, 1980; Roizin-Towle & Hall, 1981), there is no apparent *in vivo* sensitization (Rose *et al.*, 1980; Clement *et al.*, 1980; Stephens *et al.*, 1981). As noted previously (Clement *et al.*, 1980), this may be due to the fact that cis-DDP exerts its cross-linking effect via a coordination mechanism that does not involve free-radical intermediates (Rosenberg, 1979).

Since MISO is known to deplete intracellular levels of GSH in hypoxic cells (Varnes *et al.*, 1980; Taylor *et al.*, 1982*a*), we also investigated the possible involvement of glutathione in chemosensitization. Both DEM and MISO did cause a significant alteration of GSH levels (Figure 5), but this was tissue specific and possibly related to the requirement of hypoxia for MISO but not for DEM to deplete GSH *in vitro* (Varnes *et al.*, 1980). Although the enhancement of MEL cross-linking by DEM in both spleen and tumour (by a factor of 2.0 and 1.8, respectively, see Table III) may be related to GSH depletion, the lack of GSH depletion by MISO in spleen despite the observed enhancement of MEL by MISO (by a factor of 1.5) in this tissue suggests that, while GSH depletion may play a part in chemosensitization, it is unlikely to account for the whole effect. Although MISO has been found to deplete GSH in hypoxic cells, depletion could only account for part (15–20%) of the increased cytotoxicity of MEL (Taylor *et al.*, 1982*a*., *b*). The lack of GSH depletion by MISO in spleen is probably indicative of a relative absence of hypoxic cells therein.

In summary, we have shown that pretreatment with misonidazole increases drug-induced cross-linking in tissues *in vivo* after subsequent exposure to alkylating agents such as melphalan. However, because of the relatively slow rates of cross-link formation and removal with this drug, it is difficult to discriminate between several possible mechanisms for this effect. Studies using drugs with which cross-link formation is expressed almost immediately *in vitro,* such as mitomycin C (Meyn *et al.*, 1982) or nitrogen mustard (Ross *et al.*, 1978) are currently being investigated so that events occurring at much earlier times may be observed *in vivo.*

This investigation was supported by PHS grants CA 26312 and CA 23270 awarded by the National Cancer Institute, DHSS. We thank Tim Jenkins for his excellent technical assistance, and Susan Jenkins for her discussion and reading of the manuscript.

### References

BUMP, E.A., YU, N.Y. & BROWN, J.M. (1982). The use of drugs which deplete intracellular glutathione in hypoxic cell radiosensitization. *Int. J. Radiat. Oncol. Biol. Phys.*, **8**, 439.

CESARONE, C.F., BOLOGNESI, C. & SANTI, L. (1979). Improved microfluorimetric DNA determination in biological material using 33258 Hoechst. *Anal. Biochem.*, **100**, 188.

CLEMENT, J.J., GORMAN, M.S., WODINSKY, I., CATANE, R. & JOHNSON, R.K. (1980). Enhancement of antitumour activity of alkylating agents by the radiation sensitizer misonidazole. *Cancer Res.*, **40**, 4165.

CLUTTERBUCK, R.D., MILLAR, J.L. & MCELWAIN, T.J. (1982). Misonidazole enhancement of the action of BCNU and melphalan against human melanoma xenografts. *Am. J. Clin. Oncol.*, **5**, 73.

FOWLER, J.F. (1982). Workshop on the enhancement of chemotherapy by nitroimidazoles. *Br. J. Cancer*, **45**, 158.

GURTOO, H.L., HIPKENS, J.H. & SHARMA, S.D. (1981). Role of glutathione in the metabolism-dependent toxicity and chemotherapy of cyclophosphamide. *Cancer Res.*, **41**, 3584.

HISSIN, P.J. & HILF, R. (1976). A fluorimetric method for determination of oxidized and reduced glutathione in tissues. *Anal. Biochem.*, **74**, 214.

HORSMAN, M.R., BROWN, J.M. & SCHELLEY, S.L. (1982). The effect of misonidazole on the cytotoxicity and repair of potentially lethal damage from alkylating agents *in vitro*. *Int. J. Radiat. Oncol. Biol. Phys.*, **8**, 761.

KLEIN, L., PRESANT, C.A., VOGEL, C.L., GAMS, R, & JOHNSON, R. (1982). Phase 1 study of misonidazole and cyclophosphamide in solid tumours. *Int. J. Radiat. Oncol. Biol. Phys.*, **8**, 809.

KOHN, K.W. (1979). DNA as a target in cancer chemotherapy: Measurement of macromolecular DNA damage produced in mammalian cells by anticancer agents and carcinogens. *Methods Cancer Res.*, **16**, 291.

KOHN, K.W., ERICKSON, L.C., EWIG, R.A.G. & FRIEDMAN, C.A. (1976). Fractionation of DNA from mammalian cells by alkaline elution. *Biochemistry*, **15**, 4629.

LAW, M.P., HIRST, D.G. & BROWN, J.M. (1981). Enhancing effect of misonidazole on the response of the RIF-1 tumour to cyclophosphamide. *Br. J. Cancer*, **44**, 208.

MARTIN, W.M.C., MCNALLY, N.J. & DERONDE, J. (1981). Enhancement of the effect of cytotoxic drugs by radiosensitizers. *Br. J. Cancer*, **43**, 756.

MEYN, R.E., JENKINS, S.F. & THOMPSON, L.H. (1982). Defective removal of DNA cross-links in a repair-deficient mutant of Chinese hamster cells. *Cancer Res.*, **42**, 3106.

MCNALLY, N.J. (1982). Enhancement of chemotherapy agents. *Int. J. Radiat. Oncol. Biol. Phys.*, **8**, 593.

MILLAR, B.C. (1982). Hypoxic cell radiosensitizers as potential adjuvants to conventional chemotherapy for the treatment of cancer. *Biochem. Pharmacol.*, **31**, 2439.

MULCAHY, R.T., SIEMANN, D.W. & SUTHERLAND, R.M. (1981). *In vivo* response of KHT sarcomas to combination chemotherapy with radiosensitizers and BCNU. *Br. J. Cancer*, **43**, 93.

RIMONDI, C., BUSUTTI, L. & BRECCIA, A. (1982). Clinical trial of maintenance therapy with cyclophosphamide vs misonidazole and cyclophosphamide in patients with non oat cell unoperable lung carcinoma already treated with misonidazole and radiation. *Int. J. Radiat. Oncol. Biol. Phys.*, **8**, 809.

ROIZIN-TOWLE, L.A. & HALL, E.J. (1981). Enhanced cytotoxicity of antineoplastic agents following prolonged exposure to misonidazole. *Br. J. Cancer*, **44**, 201.

ROIZIN-TOWLE, L., HALL, E.J., FLYNN, M., BIAGLOW, J.E. & VARNES, M.E. (1982). Enhanced cytotoxicity of melphalan by prolonged exposure to nitroimidazoles: The role of endogenous thiols. *Int. J. Radiat. Oncol. Biol. Phys.*, **8**, 757.

ROSE, C.M., MILLAR, J.L., PEACOCK, J.H., PHELPS, T.A. & STEPHENS, T.C. (1980). Differential enhancement of melphalan cytotoxicity in tumour and normal tissue by misonidazole. In *Radiation Sensitizers: Their use in the clinical management of cancer*. (Ed. Brady). New York: Masson, p. 250.

ROSENBERG, B. (1979). Anticancer activity of cis-dichlorodiammine platinum (II) and some relevant chemistry. *Cancer Treat. Rep.* **63**, 1433.

ROSS, W.E., EWIG, R.A.G. & KOHN, K.W. (1978). Differences between melphalan and nitrogen mustard in the formation and removal of DNA cross-links. *Cancer Res.*, **38**, 1502.

SIEMANN, D.W. & MULCAHY, R.T. (1982). Cell survival recovery kinetics in the KHT sarcoma following treatment with five alkylating agents and misonidazole. *Int. J. Radiat. Oncol. Biol. Phys.*, **8**, 619.

STEPHENS, T.C., COURTENAY, V.D., MILLS, J., PEACOCK, J.H., ROSE, C.M. & SPOONER, D. (1981). Enhanced cell killing in Lewis Lung carcinoma and a human pancreatic-carcinoma xenograft by the combination of cytotoxic drugs and misonidazole. *Br. J. Cancer*, **43**, 451.

STRATFORD, I.J., ADAMS, G.E., HORSMAN, M.R. & 4 others. (1980). The interaction of misonidazole with radiation, chemotherapeutic agents, or heat. *Cancer Clin. Trials*, **3**, 231.

SUIT, H.D. & SUCHATO, C. (1967). Hyperbaric oxygen and radiotherapy of a fibrosarcoma and of a squamous-cell carcinoma of C3H mice. *Radiology*, **89**, 713.

SUTHERLAND, R.M. (1974). Selective chemotherapy of noncycling cells in an *in vitro* tumor model. *Cancer Res.*, **34**, 3501.

TANNOCK, I.F. (1980). *In vivo* interaction of anti-cancer drugs with misonidazole or metronidazole: cyclophosphamide and BCNU. *Br. J. Cancer*, **42**, 871.

TAYLOR, Y.C., BUMP, E.A. & BROWN, J.M. (1982a). Studies on the mechanism of chemosensitization by misonidazole *in vitro*. *Int. J. Radiat. Oncol. Biol. Phys.*, **8**, 705.

TAYLOR, Y.C., EVANS, J.W. & BROWN, J.M. (1982b). Mechanism of sensitization by hypoxic pretreatment with misonidazole. *Radiat. Res.*, **91**, 379.

TWENTYMAN, P.R. (1981). Modification of tumour and host response to cyclophosphamide by misonidazole and by WR 2721. *Br. J. Cancer*, **43**, 745.

VARNES, M.E., BIAGLOW, J.E., KOCH, C.J. & HALL, E.J. (1980). Depletion of non-protein thiols of hypoxic cells by misonidazole and metronidazole. In *Radiation Sensitizers: Their use in the clinical management of cancer*. (Ed. Brady) New York: Masson, p. 121.

WANG, Y-M., MADANAT, F.F., KIMBALL, J.C. & 4 others. (1980). Effect of vitamin E against adriamycin-induced toxicity in rabbits. *Cancer Res.*, **40**, 1022.

YOUNES, M. & SIEGERS, C.P. (1981). Mechanistic aspects of enhanced lipid peroxidation following glutathione depletion *in vivo*. *Chem. Biol. Interact.*, **34**, 257.